# Enhanced Crystallization of Sustainable Polylactic Acid Composites Incorporating Recycled Industrial Cement

**DOI:** 10.3390/polym16121666

**Published:** 2024-06-12

**Authors:** Yong-Min Lee, Kwan-Woo Kim, Jae-Yeon Yang, Byung-Joo Kim

**Affiliations:** 1 Research & Development Division, Korea Carbon Industry Promotion Agency, Jeonju 54852, Republic of Korea; 4ym.lee@gmail.com (Y.-M.L.); kkw1988@kcarbon.or.kr (K.-W.K.); 2 Department of Materials Science and Chemical Engineering, Jeonju University, Jeonju 55069, Republic of Korea; 3 Material Application Research Institute, Jeonju University, Jeonju 55069, Republic of Korea

**Keywords:** polylactic acid, nucleating agent, waste cement, mechanical properties, biodegradation

## Abstract

Globally, the demand for single-use plastics has increased due to the rising demand for food delivery and household goods. This has led to environmental challenges caused by indiscriminate dumping and disposal. To address this issue, non-degradable plastics are being replaced with biodegradable alternatives. Polylactic acid (PLA) is a type of biodegradable plastic that has excellent mechanical properties. However, its applications are limited due to its low crystallinity and brittleness. Studies have been conducted to combat these limitations using carbon or inorganic nucleating agents. In this study, waste cement and PLA were mixed to investigate the effect of the hybrid inorganic nucleating agent on the crystallinity and mechanical properties of PLA. Waste cement accelerated the lamellar growth of PLA and improved its crystallinity. The results indicate that the flexural and impact strengths increased by approximately 3.63% and 76.18%, respectively.

## 1. Introduction

Recently, the global demand for food delivery, packaging, and household goods has increased, leading to a rise in the use of single-use plastic products [1,2,3,4]. Common disposable plastics are mostly made of non-degradable polymer materials, and disposable plastics generated as waste are partially recycled and reused. However, most of these cause environmental challenges because of indiscriminate dumping or total disposal [5,6]. Therefore, to address these challenges, several studies have been conducted to reduce the amount of non-degradable polymers and replace them with biodegradable polymers [7,8,9,10,11]. Among these biodegradable polymers, polylactic acid (PLA), which is a semi-crystalline polymer with excellent mechanical strength and thermal processability, is typically used [12,13,14,15,16]. Owing to its excellent physical properties, PLA is widely used in the medical and food packaging fields through various molding methods such as film extrusion, injection molding, and 3D printing [17,18,19,20]. However, PLA is limited in various applications owing to its low crystallinity or slow crystallization rate, low heat distortion temperature, heat resistance, and moisture absorption [21,22]. Nevertheless, these crystallization properties can be improved using a nucleating agent (NA) [23]. Generally, talc [24,25], sodium stearate, montmorillonite [26,27], and calcium carbonate (CaCO_3_)-based NA [28,29,30,31] are used as NA. Among them, CaCO_3_-based NA is primarily used because of its low shrinkage rate and low price compared with those of other NAs.

Cement composites are used in structures such as buildings, bridges, and roads because of their excellent mechanical properties, durability, and interfacial adhesion with reinforcing bars [32,33,34,35]. Structures that are old or have reached the end of their life are being recovered and disposed of using landfill methods [36]. However, landfills require high costs in the landfill process and change to an environment where plants cannot grow because of the nature of alkaline cement [37,38]. To combat these environmental challenges, waste cement (WC) comprising inorganic compounds and CaCO_3_ was recycled as an NA in this study. As an NA, WC was mixed with PLA at various mixing ratios. Subsequently, the crystallinity, spherulite observation, and biodegradation characteristics of PLA were investigated.

## 2. Experiments

### 2.1. Materials

PLA was prepared by drying the PLA pellet (PLA-2003D, Nature Works, Minneapolis, MN, USA) in an oven at 80 °C for 24 h to minimize hydrolysis while the remaining PLA was maintained at 1% humidity in a desiccator. WC was used as an NA to improve the crystallization properties of PLA, and LAK−301 (LAK, dimethyl 5-sulfoisophthalic acid potassium salt, Takemoto oil & fat Co., Ltd., Gamagori-shi, Aichi, Japan), a commercial product, was used for comparison. First, WC was ground for 10 min using a commercial blender (7011HS, Waring, Cummings Point Road, Stanford, CA, USA) and used in powder form. Subsequently, it was finely ground using a mini ball mill (PULVERISETTE 23, Fritsch, Idar-Oberstein, Birkenfeld, Germany). The milled WC was sieved, and only that with a size of 50 µm or less was used.

### 2.2. Specimen Process

**Figure 1:**
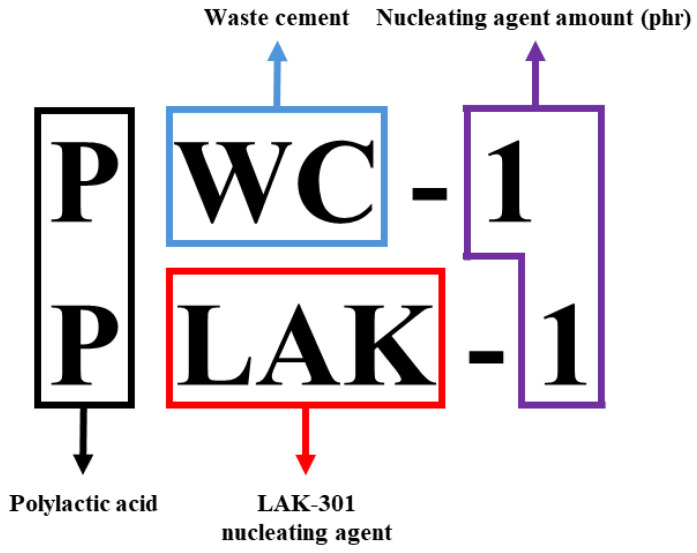
Nomenclature order of the specimens.

PLA was placed in an internal mixer heated at 180 °C and allowed to melt for 10 min. Subsequently, the NA was added (0.5–2 phr) and mixed at 50 rpm for 10 min to obtain a mixture. The mixture was then heated at 180 °C (10 °C/min) using a hot press, pressurized (50 bar) for 10 min, and then cooled at room temperature to obtain a PLA plate. The nomenclature and fabrication conditions of the specimens are presented in Table 1. Also, the nomenclature order of the specimens is presented in Figure 1.

### 2.3. Thermal Analysis of PWC

The crystallinity of PWC was measured using a differential scanning calorimeter (DSC, DSC-25, TA Instruments, New Castle, DE, USA). Approximately 5.1 ± 0.1 mg of a film was placed in a 75 µL aluminum pan, annealed within 50–200 °C in a nitrogen (50 cc/min) atmosphere, cooled within the range of 200–0 °C (−10 °C/min), and then heated within 50–200 °C (10 °C/min). The heat resistance of PLA and PWC were measured using a simultaneous thermal analyzer (TGA, TGA/DSC 3+, Mettler Toledo, Columbus, OH, USA). Approximately 5.1 ± 0.1 mg of the sample was placed in a 100 µL alumina pan and heated within 50–900 °C in an N_2_ (50 cc/min) atmosphere. The initial pyrolysis temperature (IDT), maximum weight loss temperature (T_max_), thermal stability index (A*·K*), and integral pyrolysis temperature (IPDT) were calculated based on the TGA results.

### 2.4. Analysis of PWC Crystallites

The crystallinity of PLA and PWC were analyzed using X-ray diffraction (XRD, MiniFlex, Rigaku, Tokyo, Kanto, Japan). XRD analysis was carried out with CuKα radiation (1.5406 Å) in the range of 5–60° at a scanning rate of 2°/min.

The crystallites of PLA and PWC were observed using polarized optical microscopy (POM, BX53M, Olympus, Tokyo, Kanto, Japan). The specimen was prepared via precision machining and polishing in a rectangular parallelepiped shape (L × W × T; 20 × 20 × 2 mm). The magnification of the POM used was within 50–100×, and the polarization angle was 100°.

The spherulite and lamellar of PLA and PWC were observed using atomic force microscopy (AFM, Multimode-8, Bruker, Billerica, MA, USA). The specimen was prepared via precision machining in a rectangular parallelepiped shape (L × W × T; 10 × 10 × 1 mm). The AFM used was conducted in a tapping mode.

### 2.5. Moisture Drying Test of PWC

The moisture drying amount of PLA and PWC was prepared via the precision machining of specimens in the shape of a rectangular parallelepiped (L × W × T; 62 × 25 × 3 mm). The prepared sample was subjected to moisture absorption for 7 d in a constant temperature/humidity chamber (60 °C) with a humidity of 50%, and then, the specimen was dried in an oven at 80 °C for 12 h. The mass of the dried specimen was measured using a balance and expressed as a percentage.

### 2.6. Mechanical Analysis of PWC

The flexural strengths of PLA and PWC were measured via precision processing of the rectangular parallelepiped specimens (L × W × T; 62 × 25 × 3 mm) according to ASTM D790 [39]. The test to determine the bending properties was conducted five times for each specimen via a three-point bending method using a universal testing machine (UTM, Salt, ST-1001, Incheon, Gyeonggi-do, Republic of Korea). The span distance was fixed at 48 mm, and the cross-head speed was set at 1 mm/min.

The impact strengths of PLA and PWC were prepared by precision machining the specimen (L × W × T; 63 × 13 × 3 mm; notch depth of 4 mm) into a rectangular parallelepiped shape according to ASTM D256 [40]. The impact test was conducted five times for each specimen with an Izod impact tester (RESIL Impactor, Instron, Norwood, MA, USA) using a 1.0 J hammer.

After analyzing the mechanical properties of PLA and PWC, the stress propagation shape of each specimen fracture surface was observed using scanning electron microscopy (SEM, AIS2100C, Seron Technology, Uiwang, Gyeonggi-do, Republic of Korea). To prevent the charging phenomenon, sputtering was performed for 3 min with platinum. All images were acquired at 1.0 × 10^−5^ torr and 25 kV voltage.

### 2.7. Biodegradation of PWC

**Figure 2:**
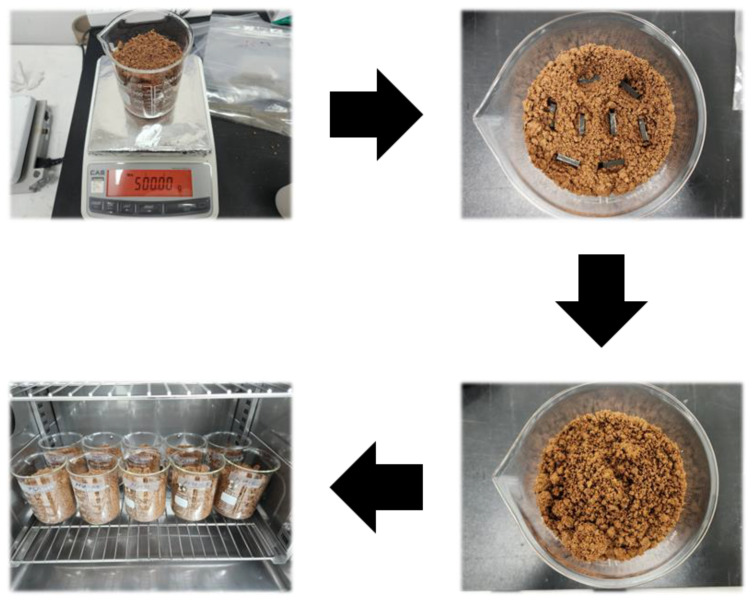
Biodegradation test process of polylactic acid/waste cement composites with a thermos-hygrostat.

The biodegradability tests of PLA and PWC were conducted using a thermo-hygrostat (TH3-PE-100, Jeio Tech, Daejeon, Chung-cheong bukdo, Republic of Korea). The specimen used in the test was produced by precision machining the specimen in a rectangular parallelepiped shape (L × W × T; 62 × 25 × 3 mm); the soil used was supplied by Gochang-gun, Jeollabuk-do, and soil 10 cm above the ground with a high density of microorganisms was used. In the biodegradation test, 500 g of soil was equally placed in a 1 L beaker, and eight specimens were used for each condition. The tests were conducted for approximately 180 d at a humidity of 50% and a temperature of 60 °C, and the mass of the specimen was verified every 30 d. Figure 2 shows the biodegradation experiment process conducted in this study.

## 3. Results and Discussion

### 3.1. Crystallite Properties with DSC

**Figure 3:**
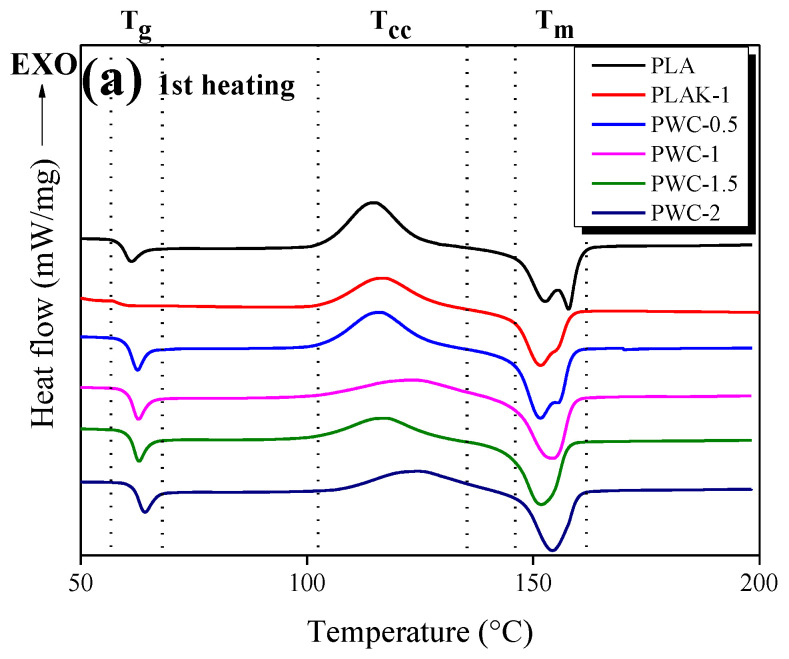

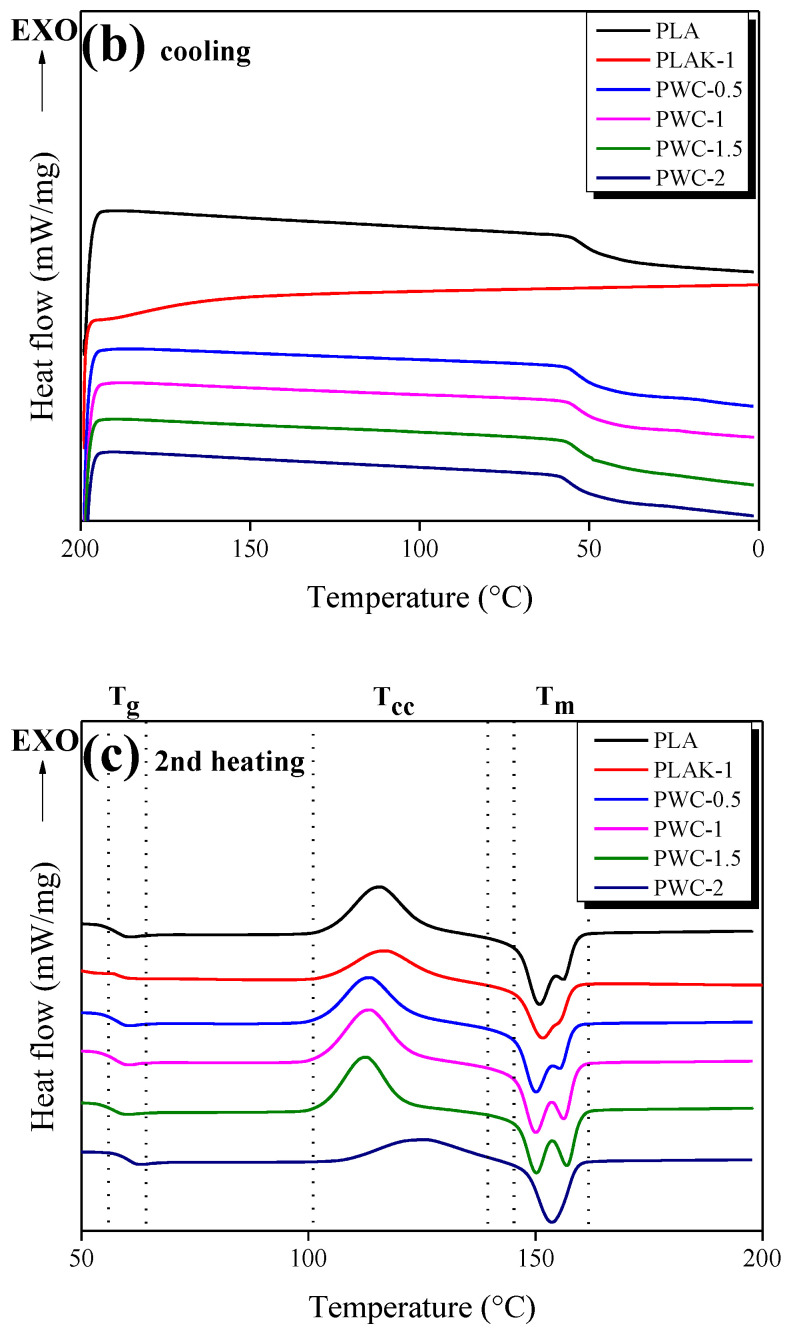
Differential scanning calorimetry thermograms of polylactic acid/waste cement composites with different waste cement contents; (**a**) first heating cycle, (**b**) cooling, and (**c**) second heating cycle.

The results of the DSC analysis of PWC mixed with various contents of WC are shown in Figure 3. The crystallinity (X_c_) of each sample was calculated using Equation (1) [41,42]. The results are shown in Table 2.

(1) $$X_{c}=\frac{{∆H}_{m}-∆H_{c}}{{∆H}_{m}^{0}}\times 100\;(\%)$$

where ${∆H}_{m}$ is the melting enthalpy (J/g), ${∆H}_{c}$ is the crystallization enthalpy, and ${∆H}_{m}^{0}$ is the melting enthalpy (93.6 J/g) when the crystallinity of PLA is 100%.

The glass transition temperature (T_g_) of the first heated sample (Figure 3a) tended to increase to approximately within the range of 61.07–64.30 °C as the WC content increased; the cold crystalline temperature (T_cc_) also increased as the WC content increased. Notably, the temperature range was widened. In addition, the melting temperature (T_m_) peak was divided into two in the case of the PLA, PLAK−1, and PWC−0.5 samples. However, the Tm peak was merged into one as the WC content increased. This is because the lamellar structure is less laminated and divided as the crystallization time is insufficient owing to the nature of PLA, which has a slow crystallization rate. However, the high content of WC improves the crystallinity of PLA and appears as a single T_m_. After annealing through first heating, the DSC result of the cooled sample (Figure 3b) exhibited no crystalline temperature (T_c_) but only T_g_ within the range of 58.89–55.38 °C. Notably, T_c_ did not appear because the time for PLA to crystallize was insufficient. On the other hand, the T_g_ of the PLAK−1 sample was observed to have lower enthalpy compared to the PWC sample during the first heating process, and it was confirmed that the T_g_ disappeared during cooling. This is believed to be because the amorphous part of PLA crystallized in the first heating process and the T_g_ disappeared. The T_g_ of the sample subjected to secondary heating (Figure 3c) was found to be approximately within 56.75–59.98 °C; the enthalpy was less than that of the T_g_ of the first heating process. In addition, the T_g_ of the PLAK−1 sample disappeared during secondary heating, and the enthalpy of T_m_ was observed to be low. T_cc_ and T_m_ were divided into two sharp peaks and two peaks from the PLA to PWC−1.5 samples, respectively, while the PWC−2 sample exhibited T_cc_ and single-peak T_m_ over a wide temperature range. Notably, WC does not improve the slow crystallization rate of PLA, although the high content of WC can improve the crystallinity of PLA. Finally, X_c_ calculated using equation 1 increased to approximately 10% as the content of WC increased. Although WC did not improve the slow crystallization rate of PLA, it showed a great improvement in crystallinity compared to LAK−301.

### 3.2. Crystallinity Properties with XRD

**Figure 4:**
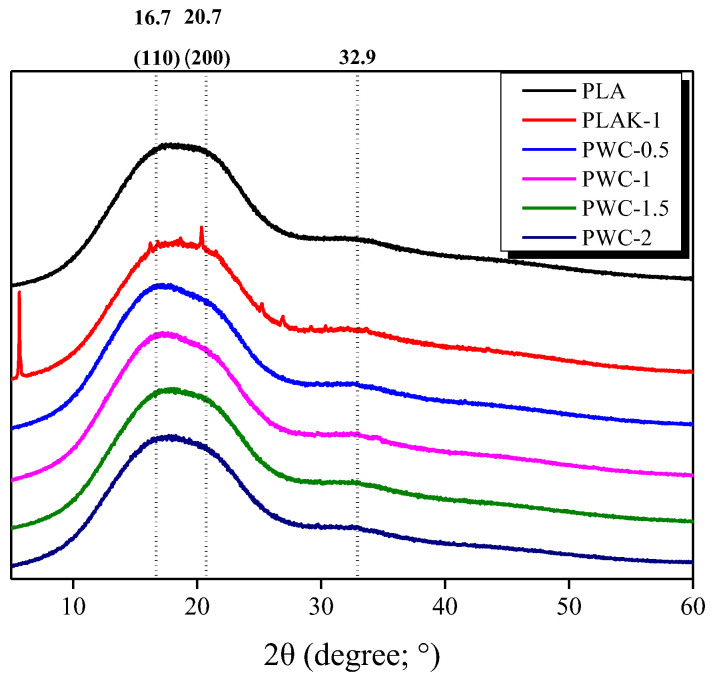
X-ray diffraction patterns of polylactic acid/waste cement composites with different waste cement contents.

The XRD spectra of PLA and PWC composites are shown in Figure 4. The strongest XRD peaks (110) and (200) were observed at scattering angles of 16.7° and 20.7°, respectively. Additionally, a broad peak was observed at a scattering angle of 32.9°, which is characteristic of PLA. The XRD peaks were observed in a broad form overall and were judged to be amorphous. However, it was confirmed that the intensity of the peaks increased with the addition of the nucleating agent. For the PLAK−1 specimen, the peak corresponding to the LAK−301 nucleating agent was observed, whereas the peak for the WC nucleating agent used in the PWC specimen was observed weakly. The XRD peaks for cured WC are shown in Figure S2 in the Supplementary Materials.

### 3.3. Thermal Stability with TGA

The IDT, T_max_, A*·K*, and IPDT of PLA and PWC were calculated using Equations (2)–(4). The results are shown in Table 3. IDT refers to the temperature at which the weight decreases more than 5% for the first time. IPDT refers to the total heat required from the initiation to the termination of decomposition. For A*·K* and IPDT, quantitative values obtained as an area ratio during single and multi-step decompositions reported by Doyle [43] were used [44].

(2) $$IPDT=A^{*}{\cdot K}^{*}\left(T_{f}{-T}_{i}\right)+T_{i}\;({}^{\circ}C)$$

(3) $$A^{*}=\frac{S_{1}+S_{2}}{S_{1}+S_{2}+S_{3}}\;({}^{\circ}C)$$

(4) $$K^{*}=\frac{S_{1}+S_{2}}{S_{1}}\;({}^{\circ}C)$$

where $A^{*}$ is the ratio of the down area of the curve to the total area of the TGA thermogram, $K^{*}$ is the coefficient of A*, $T_{i}$ (°C) is the initial temperature, and $T_{f}$ (°C) is the final temperature.

**Figure 5:**
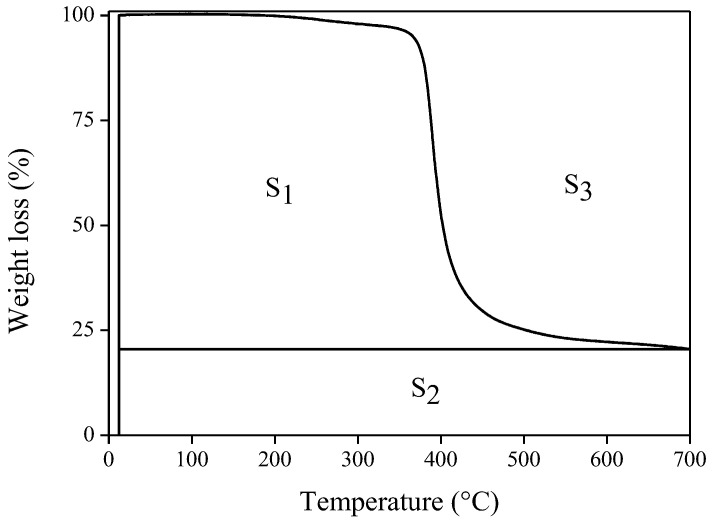
Schematic representation of S_1_, S_2_, and S_3_ for A* and K*.

Each area in the TGA thermogram is illustrated in Figure 5. A* can be expressed as the ratio of the total area of the TGA thermogram and the area of the graph. K* (a coefficient of A*) can be expressed as the ratio of the total down area of the curve and the subtracted value (the total down area minus the yield area). A*·K* is a thermal stability index, with a larger value indicating higher thermal stability [44].

**Figure 6:**
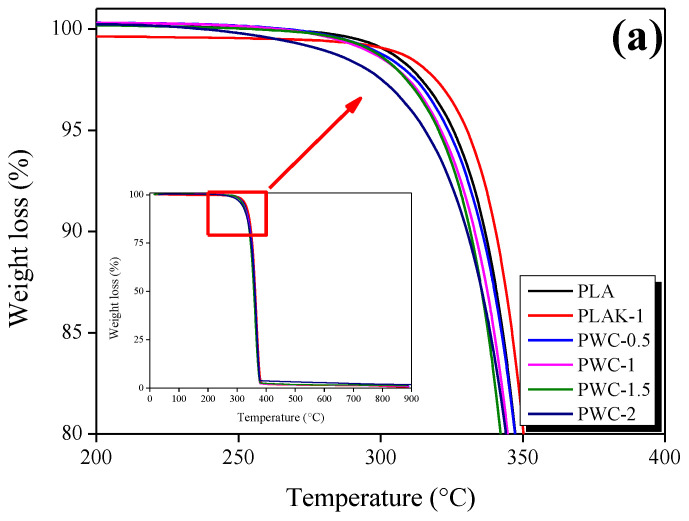

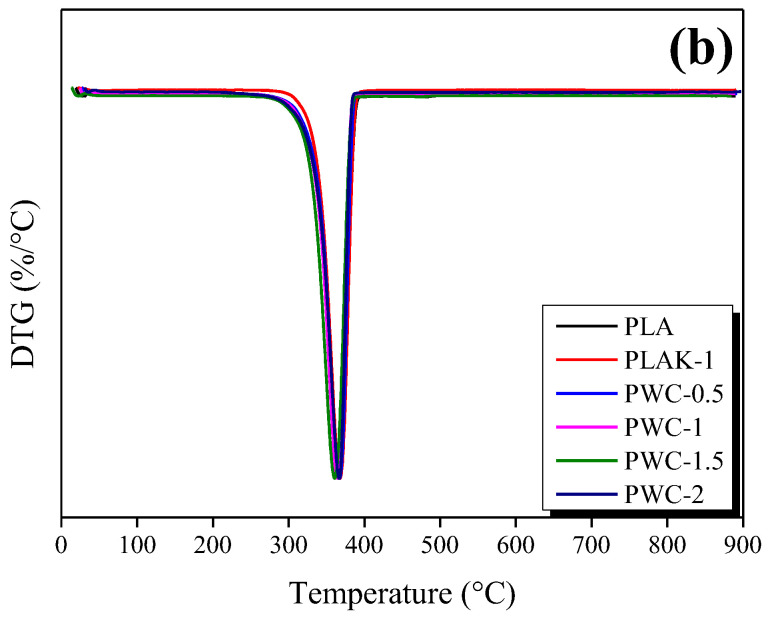
Thermogravimetric analysis and derivative TG thermograms of polylactic acid/waste cement composites analyzed as a function of waste cement content in and nitrogen atmosphere: (**a**) TGA and (**b**) DTG.

The results of the TGA and DTG analyses of PLA and PWC are shown in Figure 6a,b. All samples exhibited one thermal decomposition at approximately 320 °C. The PLAK−1 specimen exhibited a weight loss of 0.5 wt.% up to about 262 °C, followed by a rapid weight loss. On the other hand, the PWC specimens showed rapid weight loss from about 227 °C. This is considered the thermal decomposition of calcium hydroxide produced in the hydration reaction of cement. The heat resistance of all specimens was confirmed to be similar. However, the heat resistance did not improve because the CSH gel generated from cement curing was decomposed within 560–800 °C.

### 3.4. Spherulite and Lamellar Structure Analysis

**Figure 7:**
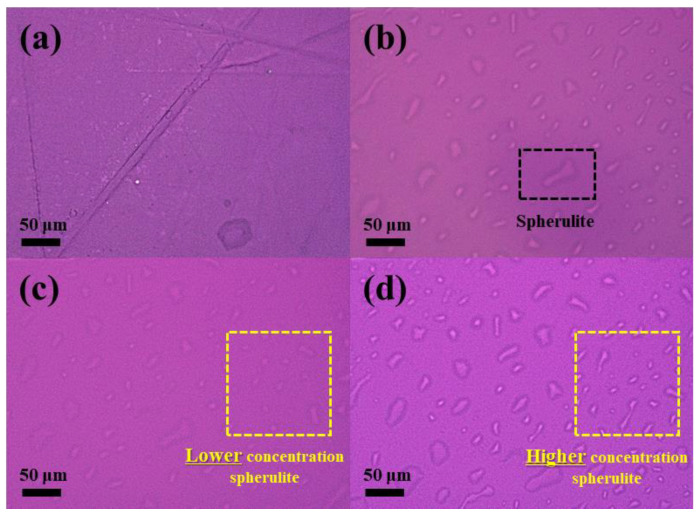
Polarized optical microscopy images of polylactic acid/waste cement composites analyzed as a function of waste cement: (**a**) PLA, (**b**) PLAK−1, (**c**) PWC−1, and (**d**) PWC−2.

The spherulite morphology images of PLA and PWC composites are shown in Figure 7. Herein, PLA had a small spherulite size and a low concentration. However, the size and concentration of spherulite increased compared with that of PLA in the sample with the addition of an NA, despite the slow crystallization rate confirmed by the DSC results. In addition, the size of spherulite was about 5–60 μm, and the same was observed for PLAK−1, PWC−1, and PWC−2 specimens. Similar spherulite densities were observed for PLAK−1 and PWC−1, and PWC−2 had a higher spherulite density. This is a result of the nucleation effects of LAK−301 and WC, which act as heterogeneous nuclei and increase the size and density of spherulite [45]. The POM results are in approximate agreement with the crystallinity results of DSC, proving that WC is an effective NA for PLA.

**Figure 8:**
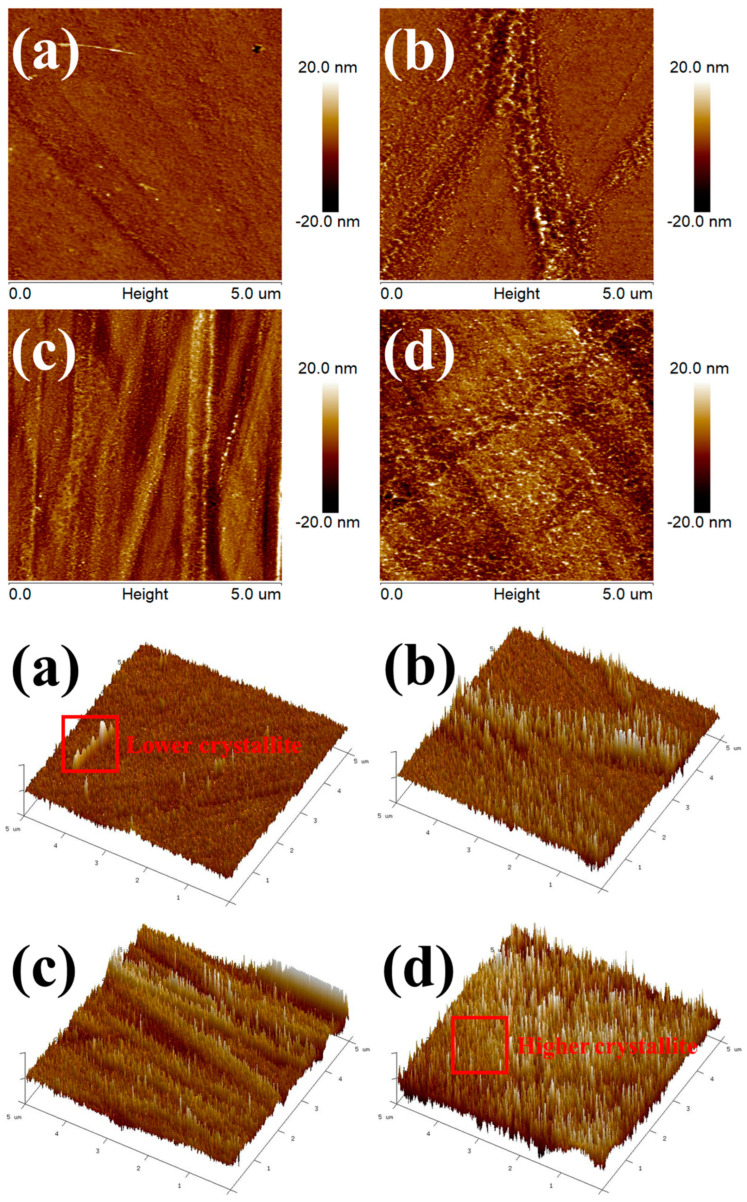
Atomic force microscopy phase images demonstrating the formation of spherulite: (**a**) PLA, (**b**) PLAK−1, (**c**) PWC−1, and (**d**) PWC−2.

The crystallite structure images of PLA and PWC composites are shown in Figure 8. The high areas (light colors) in the AFM images are the crystalline areas of each specimen. It was observed that PLA has a slow crystallization rate and is semi-crystalline and thus has low crystallinity. On the other hand, it was confirmed that the crystallinity of the PLAK−1 and PWC−1 specimens was improved compared to that of PLA. In addition, the PWC−2 was observed to have the best crystallinity due to the addition of an excessive amount of NA. Adding an excessive amount of NA induced an improvement in crystallinity, which is considered to be related to a decrease in impact strength [46].

### 3.5. Drying Properties

**Figure 9:**
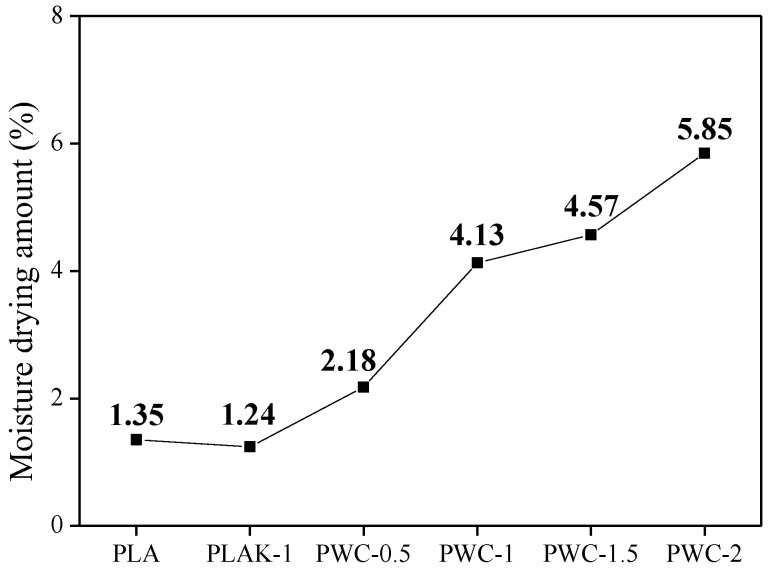
Moisture–drying properties of polylactic acid/waste cement composites.

The moisture absorption–drying test results of PLA and PWC composites are shown in Figure 9. As the content of WC increased, the drying amount of moisture inside the PLA increased. Pores may exist on the surface of cured cement, inducing rapid moisture removal during drying. The moisture drying amount of PLA and PLAK−1 was 1.35% and 1.24%, respectively, showing similar drying amounts, and LAK−301 was confirmed to have no effect on moisture drying. On the other hand, the addition of WC increased the moisture drying amount of PLA up to 5.85%. This is thought to be due to the presence of pores [47] on the surface of the cured cement, which induces rapid moisture removal during drying.

### 3.6. Mechanical Properties

**Figure 10:**
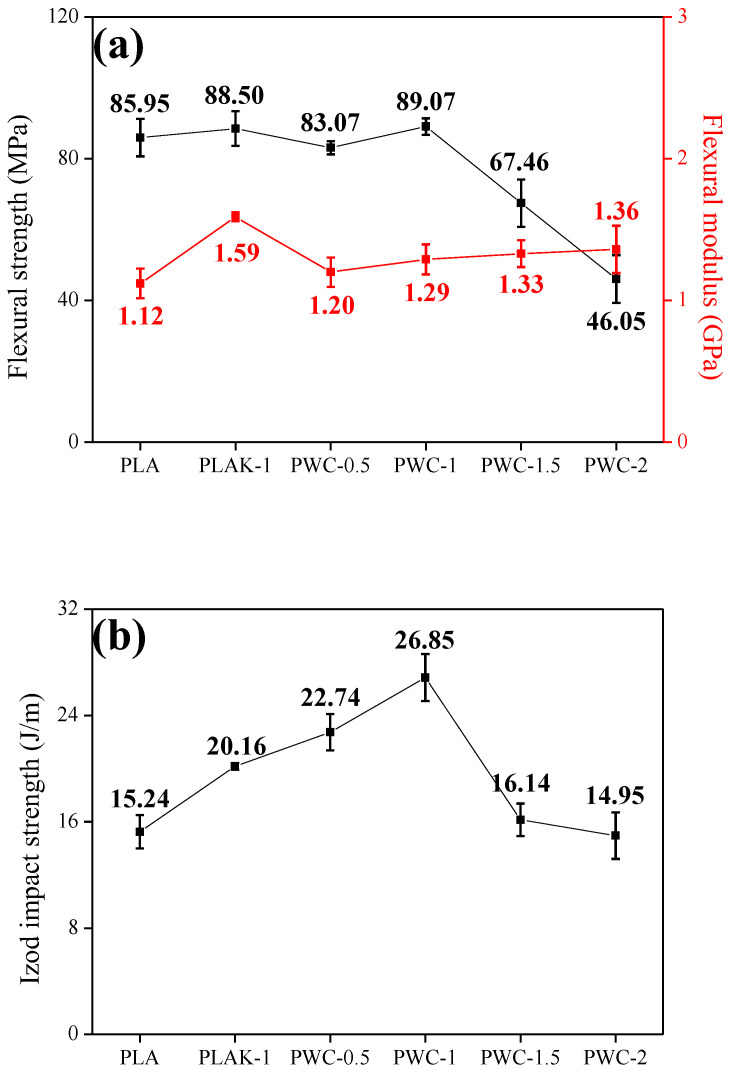
Mechanical properties of polylactic acid/waste cement composites; (**a**) flexural properties and (**b**) impact strength.

**Figure 11:**
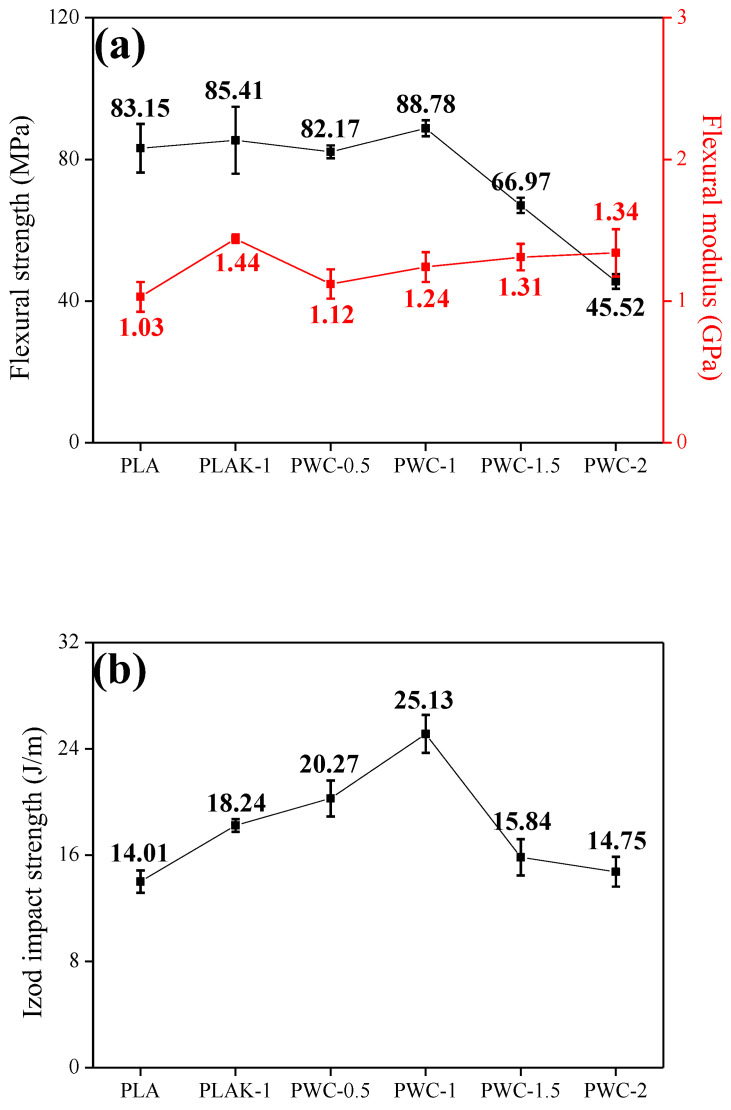
Mechanical properties of polylactic acid/waste cement composites after moisture drying test: (**a**) flexural properties and (**b**) impact strength.

The flexural and Izod impact strengths of PLA and PWC composites are shown in Figure 10 and Figure 11, respectively. In addition, Equations (5) and (6) were used to calculate the flexural and Izod impact strengths.

(5) $$S=\frac{3P_{max}L}{2bd^{2}}(Pa)$$

(6) $$S=\frac{J}{A}\;(Pa)$$

where $P_{max}$ (N) is the maximum load applied to the specimen, L (m) is the span distance of the UTM, b (m) is the length of the specimen, d (m) is the thickness of the specimen, J is the energy of the hammer used, and A (m) is the area of the specimen excluding the notch.

The flexural strength of PLA and PWC increased by about 3.63% from 85.95 MPa (PLA) to a maximum of 89.07 MPa (PWC−1). In addition, it was confirmed that the flexural modulus increased by about 21.43% from 1.12 GPa (PLA) to a maximum of 1.36 GPa (PWC−2).

The impact strength values of PWC also exhibited a similar trend. PLA was calculated to be 15.24 J/m and increased to a maximum of 26.85 J/m under the PWC−1 condition. The mechanical properties of polymers are related to crystallinity, and the T_g_ of PLA and PWC is approximately 60 °C. At room temperature, where the analysis was conducted, no fluidity was present in molecular chains [47,48]. This effect limited the increase in flexural strength, and it was confirmed that the reduction in flexural strength was larger than the increase in flexural modulus. It was judged that the WC was agglomerated and induced specimen failure. The flexural strength of PWC was found to be higher than that of PLAK, but the flexural modulus was measured to be higher for PLAK. It was judged that the WC enhances the crystallinity of PLA, thereby increasing its flexural strength, while LAK−301 improves the flexural modulus by controlling the microstructure [49]. In addition, when the content of WC increases, a large number of microcrystals are formed, and the interaction between crystals consumes a lot of energy from external impact, which was judged to lead to an improvement in impact strength.

After the moisture absorption–drying experiment, the flexural strength of PLA and PWC decreased from 85.95 MPa (PLA) to 83.15 MPa (PLA-moisture) and the flexural modulus decreased from 1.12 GPa (PLA) to 1.03 GPa (PLA-moisture). On the other hand, the flexural strength of the PWC−2 specimen was reduced from 46.05 MPa to 45.52 MPa and the flexural modulus was reduced from 1.36 GPa to 1.34 GPa.

Notably, the moisture inside PLA, which easily absorbs moisture, acted as a defect, and the mechanical strength was reduced because energy could not be transmitted from external impact (Figure 10). However, owing to the excellent moisture drying characteristics of WC, PWC increases the amount of moisture removed from the inside of PLA even at the same drying temperature and time. Also, the mechanical properties are maintained.

**Figure 12:**
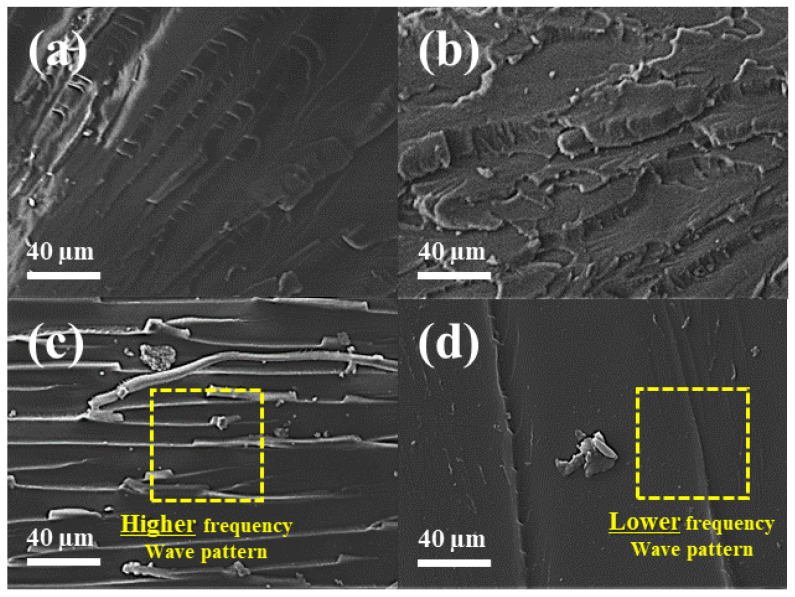
Scanning electron microscopy images of the impact fractured surface of polylactic acid/waste cement composites: (**a**) PLA, (**b**) PLAK−1, (**c**) PWC−1, and (**d**) PWC−2.

Figure 12 shows the SEM images of the fracture surface after the impact test. Compared with the fracture surfaces of PLAK−1 and PWC−1, those of PLA exhibited few wave patterns. The fracture surfaces of PLA showed some wave patterns, but the fracture surfaces of PLAK−1 and PWC−1 showed many wave patterns. On the other hand, PWC−2 was able to confirm that wave patterns were hardly visible. It was judged that PLA, PLAK−1, and PWC−1 progressed several times in the internal resistance of stress propagation in the composite material, but PWC−2 showed little resistance to stress. Notably, the internal resistance of stress propagation was improved several times in the composite material, confirming that NAs can improve the brittleness of PLA.

### 3.7. Biodegradation Properties

**Figure 13:**
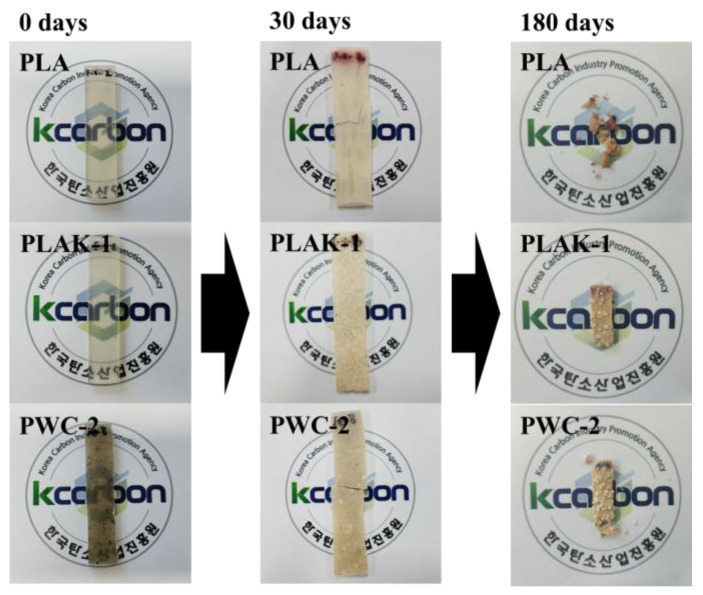
Photo images of polylactic acid/waste cement composites before biodegradation and after 180 days of biodegradation in compost at 60 °C (humidity: 50%).

**Figure 14:**
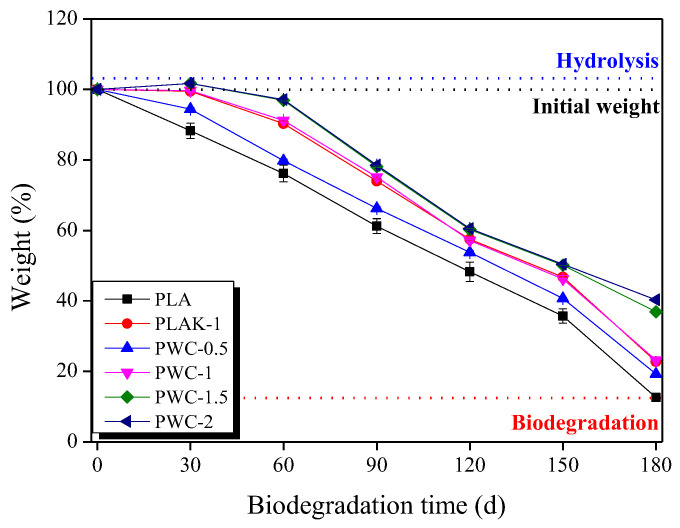
Biodegradation behavior of polylactic acid/waste cement composites.

The biodegradation images and biodegradation behaviors of PLA and PWC composites are shown in Figure 13 and Figure 14. All specimens were confirmed to exhibit surface deformation and whitening. The PLA and PWC−0.5 specimens were broken into several pieces upon contact at a short biodegradation time (15–30 d), and the remaining specimens were broken with weak force even at a long biodegradation time (45–90 d). Additionally, during the biodegradation time (120–150 d), the surface of the biodegraded specimen oligomerized and became sticky. Afterward (180 d), it was confirmed that the size of the specimen became smaller. Notably, it was hydrolyzed by microorganisms and surface whitening. Moreover, the chain of PLA broke and degraded to a relatively low molecular weight and could easily be destroyed [48]. It was judged that the crystallinity of the specimen was improved under the high content WC condition and biodegradation was delayed because the surface area for microorganisms to penetrate is small.

## 4. Conclusions

In this study, the effects of the addition of WC and changes in its content on the crystallinity and biodegradation characteristics of PLA were investigated. As the WC content increased within the range of 0.5–2 phr, the crystallization rate of PWC did not improve. However, crystallinity improved, improving flexural and impact strengths. In addition, adding WC, which exhibits excellent moisture-drying properties, simultaneously increased the amount of moisture removed during drying and temperature changes. Consequently, the mechanical strength was improved under moisture conditions. However, WC did not accelerate the biodegradation of PLA, indicating that it is an effective method to improve the crystallinity and mechanical strength of PLA/WC composites.

## Figures and Tables

**Table 1:** Nomenclature of polylactic acid/nucleating agent composites.

Nomenclature	PLA (g ^1^)	Waste Cement (phr ^2^; g ^1^)	LAK−301 (phr ^2^; g ^1^)
PLA	250	-	-
PLAK−1		-	1; 2.50
PWC−0.5	250	0.5; 1.25	-
PWC−1		1; 2.50	-
PWC−1.5		1.5; 3.75	-
PWC−2		2; 5.00	-

^1^ g: amount of material used in the experiment. ^2^ phr: parts per hundred resin (or rubber).

**Table 2:** Thermal characterization of the polylactic acid/waste cement composites with different waste cement contents.

Sample	1st T_m_ (°C)	2nd T_m_ (°C)	ΔH_m_ (J/g)	1st T_cc_ (°C)	2nd T_cc_ (°C)	X_c_ (%)
PLA	152.67/157.81	150.96/156.11	23.15/22.78	115.17	115.94	24.73/24.34
PLAK−1	151.69/155.11	151.70/155.03	25.49/29.16	117.08	116.35	27.23/31.15
PWC−0.5	151.69/155.53	150.15/155.34	26.64/30.64	116.32	113.61	28.46/32.74
PWC−1	154.53	150.11/156.21	29.71/30.68	124.22	113.71	31.74/32.78
PWC−1.5	151.85	150.20/156.89	30.78/29.22	117.72	112.92	33.88/31.22
PWC−2	154.33	153.68	31.01/31.91	125.45	125.74	33.13/34.09

**Table 3:** Thermal parameters of polylactic acid/waste cement composites analyzed in a nitrogen atmosphere.

Sample	^1^ IDT (°C)	^2^ T_max_ (°C)	^3^ A*·K*	^4^ IPDT (°C)
PLA	320.34	372.08	0.4149	375.33
PLAK−1	330.22	367.62	0.4007	371.37
PWC−0.5	323.72	370.41	0.4007	373.96
PWC−1	325.53	372.08	0.4149	380.52
PWC−1.5	321.17	370.41	0.4010	371.68
PWC−2	315.53	366.81	0.4109	387.05

^1^ IDT: initial degradation temperature. ^2^ T_max_: temperature for the maximum rate of decomposition. ^3^ A*·K*: thermal stability factor. ^4^ IPDT: integral procedural decomposition temperature.

## Data Availability

Data are contained within the article or Supplementary Materials. The original contributions presented in the study are included in the article/Supplementary Materials; further inquiries can be directed toward the corresponding authors.

## Supplementary Materials

The following supporting information can be downloaded at: https://www.mdpi.com/article/10.3390/polym16121666/s1, Figure S1: Fourier transform infrared spectra of waste cement (a) and polylactic acid/waste cement composites (b); Figure S2: Fourier transform infrared spectra of waste cement (a) and polylactic acid/waste cement composites (b).
