# Supplementary material for: Enhanced Crystallization of Sustainable Polylactic Acid Composites Incorporating Recycled Industrial Cement
Source: Polymers (Basel). 2024 Jun 12;16(12):1666. doi: 10.3390/polym16121666 (PMC11207649; doi:10.3390/polym16121666)
Supplement: Supplementary file 1 [file polymers-16-01666-s001.zip › polymers-3048076-supplementary.pdf]

Supplementary Information

Enhanced Crystallization of Sustainable Polylactic Acid Composites Incorporating Recycled Industrial Cement

Yong-Min Lee [1], Kwan-Woo Kim [1], Jae-Yeon Yang [1,*] and Byung-Joo Kim [2,3,*]

[1] Research & Development Division, Korea Carbon Industry Promotion Agency, Jeonju 54852, Republic of Korea; 4ym.lee@gmail.com (Y.-M.L.); kkw1988@kcarbon.or.kr (K.-W.K.)

[2] Department of Materials Science and Chemical Engineering, Jeonju University, Jeonju 55069, Republic of Korea

[3] Material Application Research Institute, Jeonju University, Jeonju 55069, Republic of Korea

* Correspondence: yjy1026@kcarbon.or.kr (J.-Y.Y.); kimbyungjoo@jj.ac.kr (B.-J.K.); Tel.: +82-63-219-3733 (J.-Y.Y.); +82-63-220-3293 (B.-J.K.)

Supporting table captions

Supporting Figure Captions

Figure S1. Fourier transform infrared spectra of waste cement (a) and polylactic acid/waste cement composites (b).

Figure S2. Fourier transform infrared spectra of waste cement (a) and polylactic acid/waste cement composites (b).

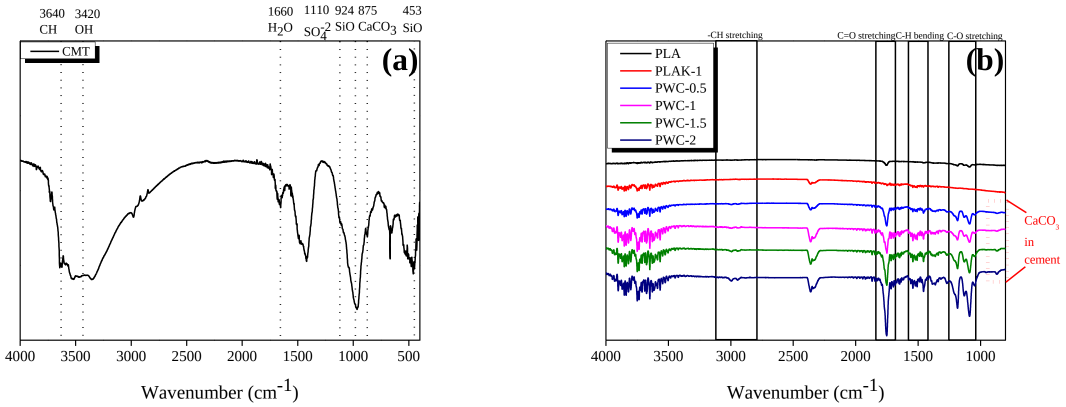

Figure S1. Fourier transform infrared spectra of waste cement (**a**) and polylactic acid/waste cement composites (**b**).

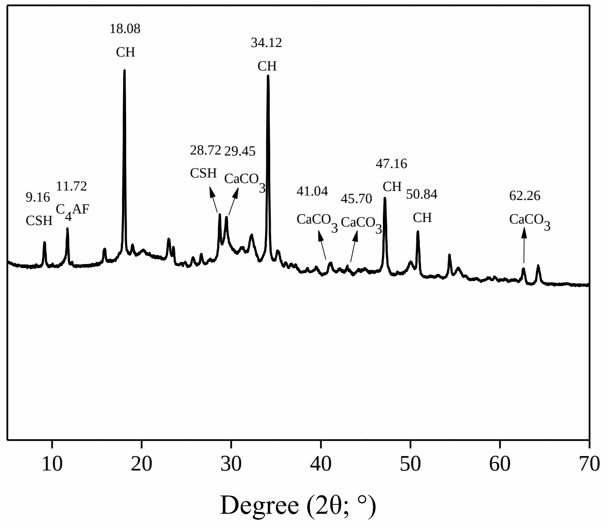

Figure S2. X-ray diffraction spectrum of waste cement.
